# Optimization of Bioactive Polyphenols Extraction from *Picea Mariana* Bark

**DOI:** 10.3390/molecules22122118

**Published:** 2017-12-01

**Authors:** Nellie Francezon, Naamwin-So-Bâwfu Romaric Meda, Tatjana Stevanovic

**Affiliations:** 1 Renewable Materials Research Centre, Department of Wood and Forest Sciences, Université Laval, Québec, QC G1V 0A6, Canada; nellie.francezon.1@ulaval.ca (N.F.); romaric.meda.1@ulaval.ca (N.-S.-B.R.M.); 2 Institute of Nutrition and Functional Food (INAF), Université Laval, Quebec, QC G1V 0A6, Canada

**Keywords:** *Picea mariana*, bark, hot water extraction, chemometrics, stilbenes, piceaside

## Abstract

Reported for its antioxidant, anti-inflammatory and non-toxicity properties, the hot water extract of *Picea mariana* bark was demonstrated to contain highly valuable bioactive polyphenols. In order to improve the recovery of these molecules, an optimization of the extraction was performed using water. Several extraction parameters were tested and extracts obtained analyzed both in terms of relative amounts of different phytochemical families and of individual molecules concentrations. As a result, low temperature (80 °C) and low ratio of bark/water (50 mg/mL) were determined to be the best parameters for an efficient polyphenol extraction and that especially for low molecular mass polyphenols. These were identified as stilbene monomers and derivatives, mainly stilbene glucoside isorhapontin (up to 12.0% of the dry extract), astringin (up to 4.6%), resveratrol (up to 0.3%), isorhapontigenin (up to 3.7%) and resveratrol glucoside piceid (up to 3.1%) which is here reported for the first time for *Picea mariana*. New stilbene derivatives, piceasides O and P were also characterized herein as new isorhapontin dimers. This study provides novel information about the optimal extraction of polyphenols from black spruce bark, especially for highly bioactive stilbenes including the *trans*-resveratrol.

## 1. Introduction

Black spruce (*Picea mariana* (Mill.) Britton, Sterns & Poggenb.) is an economically highly appreciated species of the Canadian boreal forest especially for the quality of its wood. Consequently, black spruce bark, a by-product of wood transformation, is available in large quantities. Produced at around 920,000 dry metric tons per year in Québec, spruce bark is mostly burnt to produce energy. However, this biomass rich in bioactive molecules could benefit from new alternative solution for its management. Recently identified in black spruce bark extract [1], *trans*-resveratrol, which had been studied for its multiple therapeutic properties, was found in our recent study [2] in greater concentration in black spruce bark extract (up to 667 mg/100 g of dry extract) than in common food sources such as red wine, grape skin or cocoa [3,4]. Several other active compounds identified in black spruce bark extracts such as taxifolin, pinoresinol, isolariciresinol, mearnsetin, are known for their strong antioxidant and anti-inflammatory properties [1]. Stilbenes phytoalexines isorhapontin, astringin, isorhapontigenin and piceatannol [5] were reported to be associated with antimicrobial properties, but also for therapeutic potential as antioxidant, cardioprotective, anti-inflammatory and anticancer [6,7]. Easily recoverable through simple water extraction, *trans*-resveratrol and others bioactive polyphenols could become available from black spruce bark feedstock as potential active ingredients for cosmetics, functional foods or pharmaceuticals. Moreover, acute oral toxicity of black spruce bark hot water extract obtained from our laboratory was tested on Sprague-Dawley rats on preliminary in vivo trials. With an average median lethal concentration LD_50_ toxicity greater than 2000 mg/kg, this study concluded that black spruce hot water extract showed no toxicity [8].

With the growing interest for eco-friendly chemical procedures and very strict food and cosmetics industry regulations, green processes have become of growing interest for natural ingredient extractions [9]. Among green solvents, water is of particular interest as non-toxic, non-flammable, cheap and easily available. Even if water is not the best solvent to extract active polyphenolic molecules, it still possesses remarkably flexible physico-chemical properties that can be exploited to increase the extraction selectivity [10].

Black spruce bark hot water extract has been studied as a new source of bioactive compounds, but no research has been performed so far to improve their quantity. Taking into account that purification methods are time-consuming and laborious, using rather a more selective extraction represents an interesting approach. Therefore, this study aimed to optimize the hot water extraction of bark, using factorial experimental design and high-performance liquid chromatography (HPLC) fingerprint analysis. To the best of our knowledge, this is the first study that explores the potential of chemometric based approach to determine the effects of extraction parameters on individual quantity of selected compounds from black spruce bark extract. The identification and quantification of molecules present in black spruce bark hot water extract helped us evaluate this extract as a potential health product.

## 2. Results and Discussion

### 2.1. Effects of Extraction Parameters on Multiple Response Factors

Multiple response factors (Table 1) were evaluated in this factorial design in order to understand how they were affected by extraction parameters. Factorial analysis of variance (ANOVA) was performed on the yield, the total phenolic content, total sugar content, total proanthocyanidin content and antioxidant activity of the 18 different extracts in duplicates. As for significant results, contrasts analysis was conducted in order to determine the main influencing parameter.

#### 2.1.1. Extractable Matter Yields

Highly significant in the ANOVA analysis, the extraction yield is the most affected response factors. Indeed, its value may double (from 11.2 ± 0.2% to 20.1 ± 0.7%) depending on the extraction parameters applied. The yield is one of the most important factor studied during optimization of the extraction because it determines the effectiveness of a process. In order to understand which parameters are influencing, further statistical analysis, simple contrasts, were performed (Table S1 Supplementary Material). The temperature and the ratio (bark/solvent) were demonstrated to highly affect the yield with a *p*-value below 5%. As previously reported in literature for yet another bark extraction [11], the yield is inversely proportional to the ratio (Table 1). The less the bark the larger contact area between the solvent and the raw material which decreases consequently saturation of water with extracted compounds. As for extraction temperature, applying 100 °C instead of 80 °C globally resulted in 3% increase of yields. Thus, ratio 50 mg/mL and 100 °C temperature have been considered and adopted as the best parameters for an optimized yield.

#### 2.1.2. Phenolic, Proanthocyanidin Contents and Antioxidant Capacity

As reported in multiple studies, softwood barks are very rich source of phenolic compounds, known for their health benefits [12,13]. An estimation of the total phenolic content was measured with the Folin-Ciocalteu colorimetric test, a fast and easy method, notably adapted to screening between extracts. Results demonstrated quite high polyphenol rate in black spruce bark extract, varying between 399 ± 9 mg gallic acid equivalent (GAE)/g to 502 ± 56 mg GAE/g, in accordance with previously reported studies on black spruce bark [12,14]. These results had significant differences at *p* < 5% according to ANOVA, and simple contrasts revealed that the ratio was the influencing parameter (Table S1 Supplementary Material). The lower the ratio, the higher the total phenol content (Table 1). Thus, the extraction of phenolic compounds is enhanced by the decrease of ratio. However, among phenolic compounds, the water-soluble condensed tannins (proanthocyanidins) seemed to be unaffected by extraction optimization. Results obtained for the screening of the 36 extracts on proanthocyanidin content using acidic butanol assay were relatively constant around 242 ± 6% mg cyanidin equivalent (CyE)/g of dry extract (Table 1). Indeed, no significant differences between the 18 extraction parameter combinations was reported on the ANOVA (Table S1 Supplementary Material). Thus, as polymeric phenolic compounds showed stable quantities, it seemed highly relevant to further study the variation of low molecular mass phenolic compounds in black spruce bark factorial design extraction.

Previous studies on black spruce bark hot water extract have demonstrated its important antioxidant activity [14]. Free radical scavenging activity using 2,2-diphenyl-1-picrylhydrazyl (DPPH) was screened on the 36 different extracts but no significant difference was determined according to ANOVA (Table S1 Supplementary Material). With very high values from 901 ± 174 to 1091 ± 6 µmol trolox equivalent (TE)/g dry extract, not only antioxidant activity equaled commercially available Oligopin’s (1056 ± 79 µmol TE/g dry extract) [2] but it has also been preserved regardless of the extraction parameters applied.

#### 2.1.3. Other Phytochemicals

The composition of the hot water extract of black spruce bark in terms of nitrogen and sugars contents were also investigated. Nitrogen contents in black spruce bark, which could be either from proteins or alkaloids [15], was found to be very low, around 0.24 ± 0.04% of total dry extract (data not shown). Thus, no further analysis was performed. On the other hand, soluble sugars are important constituents of spruce bark, either as free sugars or part of glycosides when linked to molecules such as polyphenols. Thus, stilbene glycosides content was reported to reach 10% of spruce bark *Picea abies* dry extract, accounting therefore for an important amount of total sugars [16]. Carbohydrates from spruce bark were reported to be mainly represented by glucose, mannose, galacturonic acid, glucuronic acid and galactose [16]. Approximate analysis of sugar content in natural extracts can be determined using the phenol-sulfuric acid method, which takes into account non-cellulosic bonded and free saccharides. In black spruce bark extracts, sugar content was determined from 534 ± 11 to 673 ± 6 mg arabinose-rhamnose-galactose-fructose equivalent (ARGF)/g dry extract (Table 1), which represents important amount. As ANOVA confirmed significant differences between the extracts, simple contrasts were conducted and revealed that ratio was once again the most influential extraction parameter (Table S1 Supplementary Material). As a matter of facts, higher ratio (200 mg/mL) appeared to be more favorable to sugar extraction (Table 1), as previously reported [17]. Interestingly, the higher the sugar content, the lower the total phenol content. This observation could be related to the greater affinity of sugar with hot water than that of less polar polyphenols [18]. Thus, a high ratio resulted in quick water saturation with carbohydrates, leaving behind the extraction of polyphenols.

### 2.2. High Performance Liquid Chromatography Fingerprint and Chemometric Analysis of Black Spruce Bark Extract Low Molecular Weight Phenolic Compounds

Fingerprint analysis with HPLC-Diode array detection (DAD) has been the method of choice for quality test or to determine the origin of herbal drugs and products [19]. In those applications, low molecular weight phenolic compounds were considered efficient descriptors of some of the product’s features, often based on polyphenolic characteristic chemical profiles [20]. Stilbene compounds can be considered chemotaxonomic features of *Picea* species. Taking into account that their maxima of absorption is around 320 nm, this wavelength was chosen for further analysis of chromatographic profiles by chemometric analysis.

**Figure 1:**
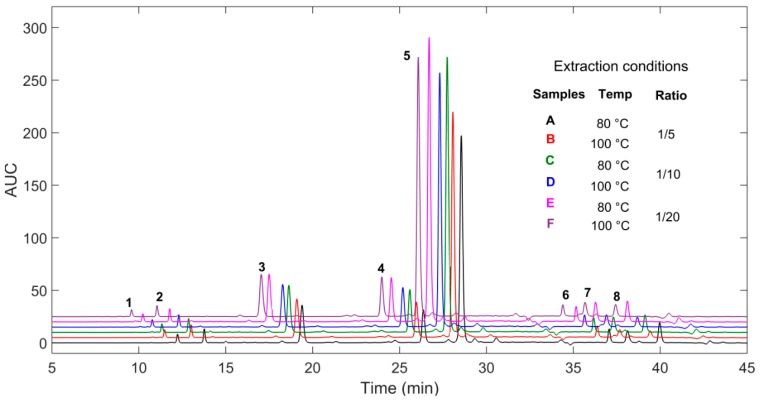
Superposed chromatogram profiles of some of the 36 extracts.

HPLC phenolic profiles of the 36 extracts are quite similar with slight quantitative differences (Figure 1). The targeted 8 main peaks exhibit essentially the same pattern in terms of concentration proportions. Hence, a chemometric analysis was performed using Principal Component Analysis (PCA) to highlight the variability of phenolic composition as a function of the extraction parameters used. PCA is a powerful tool and probably the most frequently used chemometric technique for analyzing complex multivariate measurements in chemistry. In PCA, the most important information of the dataset is condensed into synthetic linear combinations of the variables called Principal Components (PC). Their graphical representations, the component pattern and the scores plot, contribute to a better understanding and visualization of the multidimensional dataset they summarized [20].

**Figure 2:**
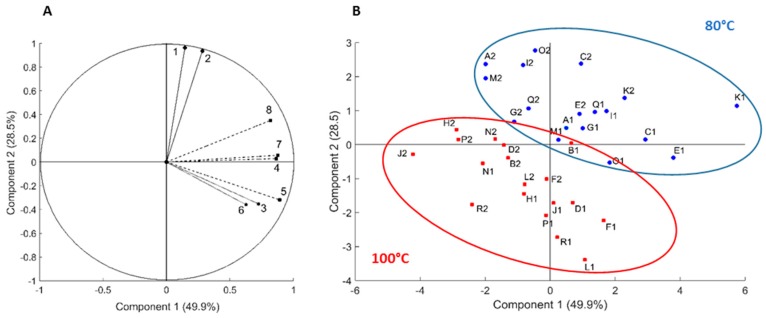
Principal component analysis of the 36 extracts of black spruce bark; (**A**) Correlation circle which displays the 8 targeted molecules correlations on the two principal components (axes); (**B**) Score plot of the 36 extracts (A1 to R2) in function of the two principal components.

The explorative analysis of the eight main monomeric compounds relative amounts in the 36 extracts revealed that the first three principal components PC1 (49.9%), PC2 (28.5%) and PC3 (8.2%) explained 86.7% of the total variance, which is highly representative of the model (Figure S1 Supplementary Material). Correlation analysis (Table S2 Supplementary Material) revealed that compounds **1** and **2** were highly correlated with PC2 (respective correlation coefficient of 0.96 and 0.93) and compounds **4**, **5**, **7** and **8** with PC1 (respective correlation coefficient 0.86, 0.89, 0.87, 0.81). The correlation circle (Figure 2A) which illustrate the variables according to the PCs, confirmed the high correlation of the compounds to both of the PCs. Compounds **3** and **6** were less explicative of the model as they were not well projected on the PCs. None of the compounds was correlated with PC3, thereby, only PC1 and PC2 (which represents 78.4% of the total variance) were retained for the analysis. The score plot (Figure 2B) which represents the distribution of the extracts according to the PC1 and PC2, revealed a division tendency of two groups as a function of temperature. Extracts produced at 80 °C seemed to gather in the upper right part (with positive coordinates for PC1 and PC2) and 100 °C extracts in the lower left part (with negative coordinates).

Moreover, the sum of variables 1 and 2 is even more correlated to PC2 that the components taken individually. As a matter of fact, compounds **1** and **2** can be gathered into a group of molecules, group A. This also applies to compounds **4**, **5**, **7** and **8** with PC1, therefore they will be considered as another group, group B. The 80 °C extracts are mostly congregated according to group A and B direction. Considering the values of the projections of the 80 °C extracts on PC1, they all demonstrated positive coordinates which means that the 80 °C extracts concentrated higher quantities of A and B groups than the extracts obtained at 100 °C.

Despite the fact that none of the extraction parameters seemed suitable to preferentially extract selected compounds from the others, PCAs, representing 76.4% of the model, allowed to highlight two groups of compounds which seemed to have the same tendencies and which were both influenced by the extraction temperature. In order to support this conclusion, the 8 compounds needed to be identified.

### 2.3. Isolation, Identification and Quantification of Low Molecular Weight Phenolic Constituents of Black Spruce Bark Extract

**Figure 3:**
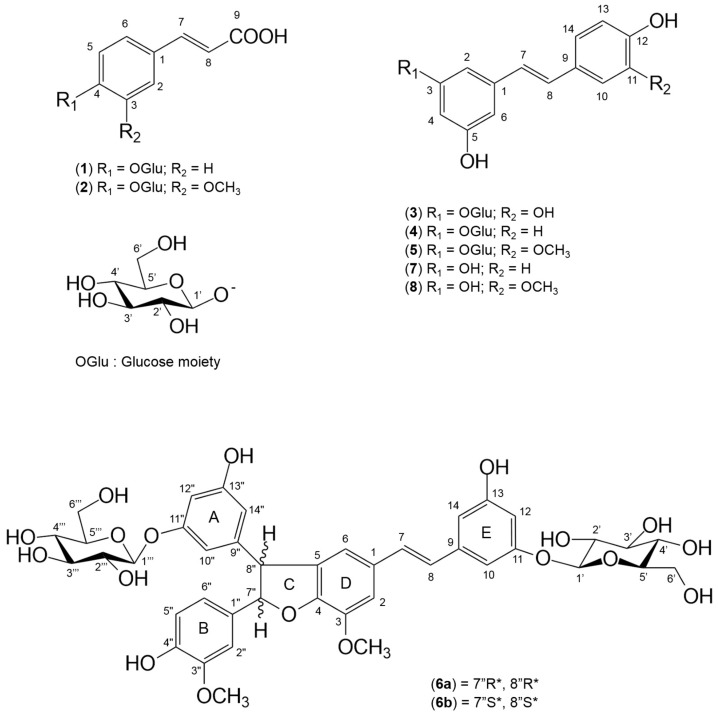
Structures of the molecules isolated from black spruce hot water extract.

The eight targeted phenolic compounds were therefore purified using silica and sephadex open columns and semi-preparative chromatography. Their characterization was achieved using a combination of ultraviolet (UV) profiles, mass spectra and nuclear magnetic resonance (NMR) shifts (Table 2). Compounds of group A were found to be glycosylated hydroxycinnamic acids, namely *trans*-*p*-coumaric acid β-d-glucopyranoside (**1**) and *trans*-ferulic acid β-d-glucopyranoside (**2**) (Figure 3). Their glucose moiety was confirmed to be linked to carbon 4 (position *para*) by nuclear overhauser spectroscopy (NOESY) analysis. The *trans*-*p*-coumaric acid, the *trans*-ferulic acid and their glycosides were previously reported in *Picea mariana* and other *Picea* species [1,21]. However, this is the first time that glucose-bound coumaric and ferulic acids (**1**) and (**2**) are reported in *Picea mariana*. Interestingly, several studies referred to these hydroxycinnamic acids as widely distributed among gymnosperms as bound to cell-wall [22]. As for the group B compounds, they are all stilbene derivatives. Molecules **4** and **5** were determined to be stilbene glycosides *trans*-piceid (**4**) and *trans*-isorhapontin (**5**) (Figure 3). To the best of our knowledge, this is the first time that *trans*-piceid was reported in *P. mariana*. Their aglycons are identified as *trans*-resveratrol (**7**) and *trans*-isorhapontigenin (**8**) (Figure 3). Even if not included in group B, compound **3** is a member of the stilbene family, identified as *trans*-astringin, a piceatannol glucoside (Figure 3).

As for the compound **6**, no match for its molar mass was found in the literature, hence further analysis were performed. Positive high resolution mass spectrometry showed a unique [M + NH_4_]^+^ ion peak at *m*/*z* = 856.3026 (originally calculated exact mass = 838.2684) from which was generated the molecular formula C_42_H_46_O_18_. High molar mass molecules from *Picea abies* had been previously reported in the literature as stilbene dimers, named piceasides [23,24]. To date, fourteen piceasides (A to N) had been identified, but none of them corresponded to a molecular mass of 838. Therefore, structural elucidation of **6** was performed using 1D (^1^H, ^13^C) and 2D NMR analyses. Interestingly, the ^13^C NMR spectrum displayed signals in doublet (Table 3), which is characteristic of a mix of stereoisomers such as diastereoisomers. Moreover, ^1^H NMR spectrum showed two sets of signals overlapped or partially overlapped, confirming the diastereoisomers hypothesis as previously demonstrated for *Picea abies* stilbene dimers by Li et al. [23]. A careful assignment of NMR shifts signals for both **6a** and **6b** is presented in details in Table 3 and confirmed with literature [23,24]. The aromatic area of the spectrum was composed of two overlapped olefinic protons [**6a** and **6b**: δ_H_ 7.03 (H7), 6.87 (H8)] with 16 Hz characteristic coupling constants of a *trans*-substituted double bond and four aromatic rings : two totally overlapping ABC spin system of each **6a** and **6b** 1,3,4-trisubstituted phenyl rings (ring B) [δ_H_ 6.93 (H2”), 6.80 (H6”), 6.79 (H5”)], two partially overlapping ABC spin system of 1,3,5-trisubstituted phenyl rings (ring A) [**6a**: δ_H_ 6.42 (H10”), 6.50 (H12”), 6.34 (H14”), **6b**: δ_H_ 6.42 (H10”), 6.50 (H12”), 6.31 (H14”)], two overlapping ABC spin system of each **6a** and **6b** 1,3,5-trisubstituted phenyl rings (ring E) [δ_H_ 6.78 (H10), 6.45 (H12), 6.62 (H14)] and two overlapping AM system of **6a** and **6b** 1,3,4,5-tetrasubstituted phenyl rings (ring D) [δ_H_ 7.11 (H2), 6.83 (H6)] (Figure 3). Spectrum area between δ_H_ 5.5 and 4.4 displayed partially overlapping AX spin system of two dehydrobenzofuran moieties (ring C) [**6a**: δ_H_ 5.43 (H7’’), 4.51 (H8’’), **6b**: δ_H_ 5.44 (H7’’), 4.50 (H8’’)] and protons at anomeric carbons of glucose moieties [**6a**: δ_H_ 4.87 (H1’), 4.79 (H1’’’), **6b**: 4.87 (H1’), 4.90 (H1’’’)]. Finally, the area between δ_H_ 3.93-3.31 with overall overlapping signals indicated the presence of four glucose moieties, two for each diastereoisomers, and two overlapping signals integrating for 6H each, characteristic of methoxyl groups. [**6a**: δ_H_ 3.95 (OCH_3_), 3.82 (OCH_3_’’), **6b**: 3.95 (OCH_3_), 3.81 (OCH_3_’’)]. Heteronuclear multiple bond correlation (HMBC) spectrum analysis confirmed their position, linked respectively to C-3 and C-3’’.

Compounds **6a** and **6b** were therefore determined to be stilbene dimers resulting from the linkage of two isorhapontin molecules, confirmed by the occurrence of 4 methoxyl groups (2 for each diastereoisomer) (Figure 3). Then, the only difference between the two diastereoisomers was their absolute stereochemistry on the two chiral carbons C-7” and C-8”. *trans* position of H7” and H8” was determined analyzing the growth rates of Rotating-frame overhauser spectroscopy (ROESY) peaks, as a function of the mixing time from 100 ms to 400 ms acquired spectrum, leading to **6a** (7” R*, 8” R*) and **6b** (7” S*, 8” S*) stereochemistry. To the best of our knowledge, this is the first time that diastereoisomeric isorhapontin dimers are reported from the plant in the literature. So far, identified piceasides reported in literature are mainly dimerization products of monomeric glycosylated stilbenes astringin and isorhapontin [23,24]. Different stilbene dimers had also been reported from other genera, such as *Gnetum* and *Vitis* [25,26,27]. In the continuation of *Picea* spp. isolated stilbene dimers denomination, compounds **6a** and **6b** will therefore be named piceasides O and P respectively.

Stilbenes are represented in limited plant genera in nature and their distribution is very genus-specific [28]. Stilbenes are widely represented in *Picea* spp. This is particularly the case with the glucosides *trans*-astringin, *trans*-isorhapontin and *trans*-piceid which had been reported in several studies on chemical composition of bark extracts of *Picea* species [5,29]. While *trans*-resveratrol was identified in several very different plants, from cocoa to grapes and also Asian herbal medicine *Polygonum cuspidatum*, *trans*-isorhapontin which is almost ubiquitous in *Picea* spp. was reported in only two species outside of *Picea* genus: the flowering plants *Veratrum taliense* and *Rheum undulatum* [28]. The absence of *trans*-isorhapontin in dietary source of stilbenoids such as wine, berries and vegetables led to very poor investigation on its potential biological activity. Indeed, except an in vitro antileukemic activity reported by Mannila and Talvitie [29], no other pharmacological study was conducted on *trans*-isorhapontin. However, with the recent identification of *trans*-isorhapontin′s aglycon, *trans*-isorhapontingenin, in a traditional Chinese herbal medicine *Gnetum cleistostachyum* with anti-cancer effects [30], several studies had since been performed on the potential anti-cancer activity of this molecule [31]. There are also studies on its activities such as cardioprotective [32] and antioxidant [33]. We report here the *Picea mariana* bark extract as a rich source of these two compounds, the *trans*-isorhapontin reaching the concentration of 11971 mg/100 g of dry extract (12.0% of the total extract) and its aglycon 3654 mg/100 g dry extract (3.7%) (Table 2). The abundance of these methoxylated stilbenes in the extract confirm the methoxylation pattern of *Picea mariana* bark polyphenols revealed previously by our group for its proanthocyanidins [34].

Pharmacological interests had risen for piceatannol since it was demonstrated to exhibit anticancer properties [35], supposedly superior to *trans*-resveratrol [36]. Very few studies considered its glucoside derivative, *trans*-astringin, for which a similar chemopreventive activity was demonstrated [35]. We found high contents of *trans*-astringin in black spruce bark extract, the maximum being 4613 mg/100 g of dry extract (4.6%) (Table 2). As for the most well-known stilbene, *trans*-resveratrol, multiple researches reported anticancer, anti-inflammatory, anti-hyperlipidemic and cardioprotective activities. Previously identified and quantified in ethyl acetate fraction of *P. mariana* bark extract [1], we are reporting here a high content of *trans*-resveratrol in the crude water extract from the optimized extraction at 302 mg/100 g of dry extract representing 0.3% of the dry bark. In addition, we determined in this study for the first time, the presence of *trans*-piceid in high concentration in *Picea mariana* bark extract reaching a maximum of 3094 mg/100 g of dry extract. Less studied than its aglycon resveratrol, *trans*-piceid is nonetheless very interesting from a pharmacological point of view as therapeutic properties were reported, namely anticancer, cardioprotective, anti-inflammatory [37] and even for the treatment of Parkinson disease [38].

## 3. Materials and Methods

### 3.1. Plant Material

Bark of black spruce was supplied by Boisaco Inc. sawmill (Quebec, QC, Canada) from debarking of logs. A voucher specimen was deposited at the Herbarium Louis-Marie of the Université Laval, Québec, Canada, with reference number QFA 0358054. In the laboratory, the raw material was separated from wood residues and lichens, washed and then air-dried at ambient temperature for 5 days. Bark was milled and sieved to select particles between 0.5 and 0.25 mm.

### 3.2. Factorial Design for Multiple Extraction Experiment

A 3 × 3 × 2 factorial experimental design was set up in order to evaluate the relationship between the response factors measured and the extraction variables. The objective was to select the best conditions for extraction optimization. The three independent variables considered were extraction time (60, 90, 120 min), ratio bark/water (200 mg/mL, 100 mg/mL, 50 mg/mL) and temperature (80 °C, 100 °C); response factors measured were extraction yield, amount of phytochemicals (total phenol content, total proanthocyanidin content, total sugar content) and antioxidant activity.

### 3.3. Extraction Procedure for Optimization Experiment

Air-dried bark (5, 10 and 20 g according to selected ratio) was extracted with 100 mL of hot distilled water under reflux and filtered with a Whatman No. 3 filter [39], then 100 mL of water was used to wash the extracted bark. Aqueous filtrate was freeze-dried with a Labconco FreeZone 12 L Console Freeze Drying System (Labconco, Kansas City, MO, USA). Each extraction was performed in duplicate.

### 3.4. Isolation and Characterization of the Hot Water Extract Constituents

Air dried black spruce bark, 150 g (corresponding to 134 g of oven dried bark), was extracted with 3 L of distilled hot water under reflux for 1 h. After filtration with a Whatman No. 3 filter paper, the extract was freeze-dried with a Labconco FreeZone 12 L Console Freeze Drying System (Labconco, Kansas City, MO USA) to yield 14.9 g of dry extract. Black spruce hot water extract powder (11.23 g) was fractionated on a silica column (63–200 µm particle size) using a mixture of methylene chloride/methanol solvents from 10 to 100% methanol. Eight fractions were obtained, from which some compounds were purified in semi-preparative HPLC: Si-1 (102.1 mg) yielded compound **8** (6.1 mg), Si-2 (707.9 mg) for compound **7** (15.3 mg), Si-4 (1222.2 mg) for compounds **4** (27.8 mg) and **5** (67.4 mg), Si-5 (1687.9) for compound **3** (86.4 mg). Fraction Si-6 (1771.7 mg) was further purified on a sephadex column, resulting in 15 sub-fractions. Compounds **1** (9.8 mg) and **2** (13.9 mg) were then isolated from fractions Si-6.1 (99.3 mg) and 6.2 (45.5 mg), and compound **6** (15.4 mg) from Si-6.8 (106.6 mg). Isolation of pure compounds was performed by HPLC on a semi-preparative Zorbax SB-C18 column (21.2 × 250 mm, 7 µm) (Agilent Technologies, Santa Clara, CA, USA) with an Agilent Technologies 1260 Infinity instrument. The flow rate was 10 mL/min of several elution gradients of water/methanol adapted to targeted molecules′ polarity. The absorbance was read at 280, 320 and 340 nm with a photodiode array detector. Identification of 8 purified compounds was achieved by compiling information from UV wavelength, mass spectra and NMR shifts.

### 3.5. Phytochemicals Assessments of the Extracts

#### 3.5.1. Total Phenol Content

Total phenol content was measured for the 36 extracts with the Folin Ciocalteu test according to the method reported by St-Pierre et al. [40]. Extract samples of 500 µL were diluted to a concentration of 100 µg/mL and reacted with 2.5 mL Folin Ciocalteu reagent (1:10 *v*/*v*) and 2.0 mL sodium carbonate aqueous solution (75 g/L) during 10 min in a 50 °C hot bath. The absorbance measured at 760 nm using a UV-visible spectrophotometer Varian Cary50 (Varian Inc., Walnut Creek, CA, USA) was used to express the phenol content in terms of gallic acid equivalent (mg GAE/g of dry extract) with a calibration curve obtained for gallic acid (*y* = 0.0227*x* − 0.0083; *R*^2^ = 0.9994).

#### 3.5.2. Proanthocyanidin Content

Proanthocyanidin content of the 36 extracts was determined by the method described by Porter et al. [41]. Each extract (1 mL, concentration of 480 µg/mL) was mixed with 200 µL of 2% ferrous ammonium sulfate (FeNH_4_(SO_4_)_2_) in HCl solution (2N), and 6 mL of n-butanol:HCl (95:5) and reacted for 50 min in a 95 °C hot bath. Proanthocyanidin content was determined by reading absorbance on a UV-Vis spectrophotometry at 550 nm and expressed with a cyanidin chloride calibration curve (*y* = 0.0298*x*; *R*^2^ = 0.9953), in cyanidin chloride equivalent CyE/g dry extract.

#### 3.5.3. Total Sugar Content

Total sugar content of 36 extracts was determined by the phenol-sulfuric acid method adapted from Albalasmeh et al. [42]. Briefly, 1 mL of extract (90 µg/mL) was mixed with 500 µL of phenol reagent (4% *m*/*v* water) and 2.5 mL of concentrated sulfuric acid (36N) and reacted for 10 min in darkness. Absorbance was read at 490 nm and sugar content was expressed with an Arabinose-Rhamnose-Galactose-Fructose calibration curve (mg ARGF/g dry extract) (*y* = 0.0097*x* + 0.022; *R*^2^ = 0.9960).

#### 3.5.4. DPPH Assay

The antioxidant activity of the 36 extracts was evaluated with the radical DPPH (2,2-diphenyl-1-picrylhydrazyl) using a method adapted from Li et al. [43]. In a 96-well plate, 100 µL of each diluted extract in methanol (100 µg/mL) reacted with 150 µL DPPH (137 µM). The reduction of the radical resulted in a color change that was recorded after 30 min on a spectrophotometer at 515 nm wavelength. Percentage of DPPH radical inhibition was calculated against a blank and expressed in Trolox Equivalent (µmol TE/g dry extract) according to the standard curve (*y* = 0.0782*x* + 0.0142; *R*^2^ = 0.9785).

### 3.6. HPLC-DAD Analysis

In order to evaluate the influence of the extraction parameters on the monomeric phenolic profile of the extract, the 36 black spruce bark extracts were tested using High Performance Liquid Chromatography-Diode Array Detector analysis. The separation was performed on an Agilent Technologies Series 1100 Instrument equipped with a reverse phase Zorbax SB-C18 column (4.6 × 250 mm, 5 µm) (Agilent Technologies, Santa Clara, CA, USA). The system was run with the following elution program, with solvents (A) 1% (*v*/*v*) formic acid in water and (B) acetonitrile: 5–15% B in 10 min, isocratic 15% B for 5 min, 15–30% B in 20 min, isocratic 30% B for 5 min, 30–40% B in 5 min, isocratic 40% B for 5 min. Then, 10 min post run at initial conditions was set for column equilibration. The flow rate was 0.7 mL/min and the column temperature, 30 °C. Black spruce extracts were injected at a concentration of 1 mg/mL, 10 µL injection volume. UV detection was set at 280, 320 and 340 nm.

### 3.7. Chemometric Analysis

In order to highlight the variability observed between the 36 studied extracts by HPLC-DAD analyses, the peak areas of main phenolic compounds were then submitted to an exploratory chemometric analysis. To achieve it, a principal component analysis was applied on the dataset made of 36 samples × 8 variables (major extract constituents) [20]. The analysis was performed with SAS software package 9.4 (SAS Institute Inc., Cary, NC, USA).

### 3.8. HRMS and NMR Analysis

High Resolution-Mass Spectrometry analyses were performed on a 6210 Time-of-Flight (Agilent Technologies, Santa Clara, CA, USA) with electrospray in positive mode (ESI+), coupled with HPLC. The ESI-TOF specifications were as follow: drying gas, 5 L/min; gas temperature, 325 °C; nebulizer pressure, 30 psig; skimmer, 65 V; capillary voltage, 4000 V and fragmentor, 175 V. Recorded from 100 to 1000 *m*/*z*, mass spectra were then analyzed on an Agilent MassHunter Qualitative Analysis software (version B.02.00, Agilent Technologies, Santa Clara, CA, USA) Nuclear magnetic resonance was performed on an Agilent 400-MR DD2 system (Agilent Technologies, Santa Clara, CA, USA), either for one-dimensional ^1^H and ^13^C NMR or two-dimensional NMR (HSQC, HMBC, COSY, NOESY and ROESY) spectroscopy. NMR shifts of isolated compounds were in accordance with literature: **1** and **2** [44]; **3**, **4**, **5** and **8** [45], **7** [46] (Supplementary Material).

Compound **6** was isolated as a dark yellow amorphous solid (15.4 mg); ${\left[\alpha \right]}_{D}^{22}$ −52.0° (c = 0.25, CH_3_OH); UV (CH_3_OH) λ_max_ (log ε): 285 (4.5), 310 (4.7), 330 (4.7) nm; Infrared IR (Attenuated total reflection ATR): 3349, 2950, 2837, 1452, 1414, 1125, 1026, 984, 667 cm^−1^; ^1^H NMR (CD_3_OD, 500 MHz), ^13^C NMR (CD_3_OD, 126 MHz), HMBC: see Table 3; Positive ESI-TOF-HRMS, *m*/*z*: 856.3026 [M + NH_4_]^+^, calculated exact mass 838.2684 for C_42_H_46_O_18_.

### 3.9. Statistical Analysis

Yield, polyphenol, proanthocyanidin, sugar and antioxidant capacity assessments were expressed as means of triplicates ± standard deviation. Data sets were submitted to factorial analysis of variance (ANOVA), and contrast analysis (planned comparisons) using SAS software 9.4 (SAS Institute Inc., Cary, NC, USA).

## 4. Conclusions

We have demonstrated in this research that the chemical composition of hot water extracts of black spruce bark was affected by extraction parameters. Proportion of different families of molecules can be preferentially targeted using different extraction parameters. Thus, the recovery of phenolic compounds is favored over sugars when a low ratio (bark/water) is applied. However, high temperatures are not favorable towards extracting the phenolic compounds of interest, even though these are advantageous to getting high yields. Hence, for an overall optimized extraction of polyphenols from black spruce bark, the most suitable parameters are 80 °C and ratio 50 mg/mL. Moreover, chemometric analysis demonstrated that specific phenolic compounds classes (hydroxycinnamic acids and stilbenes) can be targeted by selected extraction parameters. Even if none of the extraction parameters allowed for classes discrimination, the 80 °C temperature was confirmed to be the best temperature parameter for extraction of polyphenols. The chemometrics therefore represents an efficient tool to examine the influence of extraction parameters.

In addition, the great amounts of bioactive resveratrol and stilbenes′ glycosides, such as astringin and isorhapontin, which make about a quarter of the whole hot water bark extract composition, may indicate a potential application of this extract as natural health product. The presence of piceid, the resveratrol glycoside, is herein reported for the first time in *Picea mariana*. In addition, the identification of novel diastereoisomeric piceasides, a first example of isorhapontin dimerization in nature, demonstrated that work needs to be pursued on further chemical characterization of black spruce bark extract. The repeatability of the chemical composition of the studied 36 crude hot water extracts from *Picea mariana* bark, combined with its previously determined safety, makes this extract applicable as a natural health product in food additives, functional foods, cosmetic ingredients and pharmaceuticals.

## Figures and Tables

**Table 1:** Factorial design for the optimization of black spruce bark extraction.

Experimental Design				Response Factors				
Extract Name	Time (min)	Temperature (°C)	Ratio (mg/mL)	Yield (%)	Phen (mg GAE/g)	PA (mg CyE/g)	Sugar (mg ARGF/g)	Antiox (µmol TE/g)
A	60	80	200	11.3 ± 0.4	442 ± 25	224 ± 8	662 ± 77	1021 ± 54
B	60	100	200	14.3 ± 0.7	471 ± 6	252 ± 2	581 ± 3	1082 ± 29
C	60	80	100	14.5 ± 0.4	469 ± 5	236 ± 15	543 ± 49	1087 ± 19
D	60	100	100	17.7 ± 0.3	472 ± 27	254 ± 8	574 ± 7	1071 ± 14
E	60	80	50	15.6 ± 0.5	485 ± 4	252 ± 16	604 ± 24	1007 ± 101
F	60	100	50	19.3 ± 0.2	468 ± 2	264 ± 1	556 ± 21	1091 ± 6
G	90	80	200	11.5 ± 0.5	456 ± 5	245 ± 1	608 ± 71	1029 ± 52
H	90	100	200	14.6 ± 0.2	426 ± 15	232 ± 20	600 ± 13	1017 ± 64
I	90	80	100	14.5 ± 0.3	486 ± 19	249 ± 14	606 ± 9	1079 ± 9
J	90	100	100	18.1 ± 0.4	438 ± 3	232 ± 1	555 ± 25	1047 ± 30
K	90	80	50	16.1 ± 0.2	472 ± 3	246 ± 34	538 ± 24	1061 ± 11
L	90	100	50	19.4 ± 0.6	459 ± 10	243 ± 3	573 ± 37	991 ± 56
M	120	80	200	11.2 ± 0.2	464 ± 45	245 ± 3	571 ± 23	901 ± 174
N	120	100	200	14.7 ± 0.3	399 ± 9	220 ± 15	637 ± 4	1026 ± 62
O	120	80	100	15.1 ± 0.4	467 ± 35	235 ± 15	617 ± 21	962 ± 87
P	120	100	100	18.8 ± 0.4	447 ± 1	238 ± 4	673 ± 6	1049 ± 27
Q	120	80	50	16.5 ± 0.1	445 ± 5	244 ± 6	579 ± 7	1086 ± 17
R	120	100	50	20.1 ± 0.7	502 ± 56	236 ± 17	534 ± 11	1075 ± 20

Response factor results are expressed as means and standard deviations from extracts in duplicates. Statistical analysis (factorial analysis of variance (ANOVA) and contrasts) are available in Table S1 Supplementary Material. Phen: total phenol content; PA: Proanthocyanidin content; Antiox: Antioxidant capacity; GAE: Gallic Acid Equivalent; CyE: Cyanidin Equivalent; ARGF: Arabinose-Rhamnose-Galactose-Fructose equivalent; TE: Trolox Equivalent; Results are expressed on g of dry extract.

**Table 2:** Identification and quantification data for the 8 phenolic compounds isolated from black spruce bark extract.

		Identification				Quantification					
Comp.	Ret. Time (min)	λ_max_ (nm)	Exact Mass	Formula	Suggested Compound	Regression Equation	*R*^2^	Linear Range ug/mL	LOD	LOQ	Concentration Range mg/100 g Dry Extracts
**1**	12.4	290	326.0994	C_15_H_18_O_8_	*trans-p*-coumaric acid β-d-glucopyranoside	*y* = 2140.2*x* + 8.2609	0.9997	5–500	0.1	0.4	160–265
**2**	13.9	290, 315	356.1093	C_16_H_20_O_9_	*trans*-ferulic acid β-d-glucopyranoside	*y* = 1486.4*x* − 60.765	0.9987	5–1000	0.4	1.3	894–1073
**3**	20.2	325, 305	406.1243	C_20_H_21_O_9_	*trans*-astringin	*y* = 2284*x* − 441.91	0.999	5–1000	3.8	11.4	2272–4613
**4**	26.8	305, 320	390.1303	C_20_H_22_O_8_	*trans*-piceid	*y* = 3384.1*x* − 528.13	0.9971	5–500	0.2	0.6	1805–3094
**5**	29.4	325, 303, 290	420.1416	C_21_H_24_O_9_	*trans*-isorhapontin	*y* = 2944.6*x* − 265.82	0.9979	1–1000	0.6	1.9	4256–11971
**6**	37.3	330, 310, 283	838.2684	C_42_H_46_O_18_	piceaside O and P	*y* = 1056.4*x* − 329.29	0.9933	1–1000	0.4	1.2	3508–4853
**7**	38.5	305, 320	228.0796	C_14_H_12_O_3_	*trans*-resveratrol	*y* = 7520.7*x* + 43.49	0.9914	1–500	0.2	0.6	53–302
**8**	40.3	325, 303, 290	258.0892	C_15_H_14_O_4_	*trans*-isorhapontigenin	*y* = 2062.4*x* − 415.08	0.9979	5–1000	0.3	0.9	2257–3654

Comp.: compound number; Ret. time: retention time; λ_max_: maximal wavelength value; Exact mass calculated from *m*/*z* (mass-to-charge ratio); *R*^2^: coefficient of determination; LOD: Limit of detection, LOQ: Limit of quantification.

**Table 3:** Nuclear Magnetic Resonance shifts of isolated molecule **6** from black spruce bark hot water extract ^1^H and ^13^C shifts and Heteronuclear Multiple Bond Correlation (HMBC) of **6a** and **6b** molecules.

Position	δ_H_ (6a) ^a^	δ_C_ (6a) ^b^	δ_H_ (6b) ^a^	δ_C_ (6b) ^b^	HMBC (6a and 6b)
1		139.7 (*d*)		139.7 (*d*)	
2	7.11 (*br s*)	110.5 (*d*)	7.11 (*br s*)	110.4 (*d*)	C-3, 7, 6, 4
3		144.3 (*s*)		144.3 (*s*)	
4		148.0 (*d*)		148.0 (*d*)	
5		131.5 (*d*)		131.5 (*d*)	
6	6.83 (*br s*)	115.6 (*d*)	6.81 (*br s*)	115.5 (*d*)	
7	7.03 (*d*, 16.3)	128.6 (*d*)	7.03 (*d*, 16.3)	128.6 (*d*)	C-8, 1, 2, 6
8	6.87 (*d*, 16)	126.2 (*d*)	6.87 (*d*, 16)	126.2 (*d*)	C-7, 9, 1, 10, 14
9		131.9 (*d*)		131.8 (*d*)	
10	6.78 (*overlap*)	105.7 (*d*)	6.78 (overlap)	105.6 (*d*)	C-11, 8, 9, 12, 14
11		159.0 (*s*)		159.0 (*s*)	
12	6.45 (*t*, 2.2)	102.9 (*s*)	6.45 (*t*, 2.2)	102.9 (*s*)	C-10, 14, 13, 11
13		158.1 (*s*)		158.1 (*s*)	
14	6.62 (*t*, 1.7)	107.0 (*s*)	6.62 (*t*, 1.7)	107.0 (*s*)	C-13, 12, 8, 10
1′	4.87 (*overlap*)	100.4 (*s*)	4.87 (*overlap*)	100.4 (*s*)	C-11
2′	3.37–3.51 (*overlap*)	73.3 (*d*)	3.37–3.51 (*overlap*)	73.4 (*d*)	
3′	3.37–3.51 (*overlap*)	76.5 (*s*)	3.37–3.51 (*overlap*)	76.5 (*s*)	
4′	3.37–3.51 (*overlap*)	70.0 (*d*)	3.37–3.51 (*overlap*)	70.0 (*d*)	
5′	3.37–3.51 (*overlap*)	76.6 (*d*)	3.37–3.51 (*overlap*)	76.6 (*d*)	
6′	3.79 (*dd*, 12.1, 2.3)	60.9 (*d*)	3.79 (*dd*, 12.1, 2.3)	60.7 (*d*)	C-4′, 5′
1″		131.6 (*d*)		131.6 (*d*)	
2″	6.93 (*d*, 6.9)	109.4 (*d*)	6.93 (*d*, 6.9)	109.3 (*d*)	C-6″, 4″, 7″, 3″ 1″
3″		147.7 (*s*)		147.7 (*s*)	
4″		146.5 (*d*)		146.5 (*d*)	
5″	6.79 (*overlap*)	114.8 (*s*)	6.79 (*overlap*)	114.8 (*s*)	C-1″, 3″
6″	6.80 (*overlap*)	119.0 (*d*)	6.80 (*overlap*)	118.8 (*d*)	C-7″, 1″
7″	5.43 (*d*, 8.8)	94.0 (*d*)	5.44 (*d*, 8.6)	94.0 (*d*)	C-8″, 2″, 6″, 1″, 3″, 9″
8″	4.51 (*d*, 8.8)	57.6 (*d*)	4.50 (*d*, 8.6)	57.6 (*d*)	C-7″, 9″, 5, 10″, 14″
9″		143.8 (*d*)		143.6 (*d*)	
10″	6.42 (*ddd*, 3.8, 2.2, 1.5)	107.6 (*d*)	6.42 (*ddd*, 3.8, 2.2, 1.5)	107.4 (*d*)	C-11″, 12″, 8″, 14″,9″
11″		159.1 (*s*)		159.1 (*s*)	
12″	6.50 (*t*, 2.2)	102.5 (*d*)	6.50 (*t*, 2.2)	102.3 (*d*)	C-13″, 11″, 10″, 14″
13″		158.5 (*d*)		158.5 (*d*)	
14″	6.34 (*dd*, 2.2, 1.4)	109.0 (*d*)	6.31 (*dd*, 2.2, 1.4)	108.9 (*d*)	C-13″, 12″, 10″, 8″
1′′′	4.79 (*d*, 7,5)	100.8 (*d*)	4.90 (*d*, 7.6)	100.9 (*d*)	C-11″
2′′′	3.37–3.51 (*overlap*)	73.5 (*s*)	3.37–3.51 (*overlap*)	73.5 (*s*)	
3′′′	3.37–3.51 (*overlap*)	76.5 (*d*)	3.37–3.51 (*overlap*)	76.5 (*d*)	
4′′′	3.37–3.51 (*overlap*)	69.7 (*d*)	3.37–3.51 (*overlap*)	69.6 (*d*)	
5′′′	3.37–3.51 (*overlap*)	76.8 (*d*)	3.37–3.51 (*overlap*)	76.8 (*d*)	
6′′′	3.93 (*dd*, 12.1, 2.1)	61.2 (*d*)	3.91 (*dd*, 12.1, 2.1)	61.2 (*d*)	C-4″, 5″
OCH3	3.95 (*s*)	55.4 (*s*)	3.95 (*s*)	55.4 (*s*)	C-3
OCH3″	3.82 (*s*)	55.0 (*d*)	3.81 (*s*)	55.0 (*d*)	C-3″

^a^: ^1^H chemical shifts acquired at 500 MHz in CD_3_OD with multiplicities and coupling constants expressed in Hz in parenthesis; ^b^: ^13^C chemicals shifts acquired at 125 MHz in CD_3_OD with multiplicities in parenthesis. Identical chemical shifts for **6a** and **6b** might be interchangeable.

## Supplementary Materials

Supplementary Materials are available online. Statistics: Figure S1. Variance explained for the principal component analysis; Table S1. ANOVA and contrasts statistical experiments, general linear model (GLM) procedure on SAS software. Pr > F, the level of significance, has to reach 0.05 (for α = 5%) for the model to be statistically significant. Contrasts are calculated if ANOVA is significant; Table S2. Pearson correlation coefficients and p values from statistical significance testing (in italics); NMR: ^1^H and ^13^C NMR shifts of isolated molecules **1**–**5** and **7**–**8** from black spruce bark hot water extract; Figure S2. ^1^H NMR of compound **6**; Figure S3. ^13^C NMR of compound **6**; Figure S4. HSQC NMR of compound **6**; Figure S5. HMBC NMR of compound **6**.
