# Supplementary material for: Optimization of Bioactive Polyphenols Extraction from Picea Mariana Bark
Source: Molecules. 2017 Dec 1;22(12):2118. doi: 10.3390/molecules22122118 (PMC6149921; doi:10.3390/molecules22122118)

Supplementary Material

Table S1. ANOVA and contrasts statistical experiments, GLM procedure on SAS software. Pr>F, the level of significance, has to reach 0.05 (for α=5%) for the model to be statistically significant. Contrasts are calculated if ANOVA is significant.

Yield dataset

Source	DF	Sum of Squares	Mean Square	F Value	Pr > F
**Model**	17	264.8631472	15.5801851	90.48	<.0001
**Error**	18	3.0995500	0.1721972		
**Corrected Total**	35	267.9626972			

R-Square	Coeff Var	Root MSE	yield Mean
0.988433	2.639783	0.414967	15.71972

Source	DF	Type I SS	Mean Square	F Value	Pr > F
**time**	2	1.6524056	0.8262028	4.80	0.0214
**ratio**	2	153.7944056	76.8972028	446.56	<.0001
**time*ratio**	4	0.6274611	0.1568653	0.91	0.4786
**temp**	1	108.0560250	108.0560250	627.51	<.0001
**time*temp**	2	0.2605500	0.1302750	0.76	0.4836
**ratio*temp**	2	0.2668167	0.1334083	0.77	0.4756
**time*ratio*temp**	4	0.2054833	0.0513708	0.30	0.8752

Polyphenol content dataset

Source	DF	Sum of Squares	Mean Square	F Value	Pr > F
**Model**	17	19374.38000	1139.66941	2.39	0.0373
**Error**	18	8572.19000	476.23278		
**Corrected Total**	35	27946.57000			

R-Square	Coeff Var	Root MSE	poly Mean
0.693265	4.750446	21.82276	459.3833

Source	DF	Type I SS	Mean Square	F Value	Pr > F
**time**	2	1335.255000	667.627500	1.40	0.2718
**ratio**	2	5180.901667	2590.450833	5.44	0.0142

**time*ratio**	4	558.558333	139.639583	0.29	0.8785
**temp**	1	1181.067778	1181.067778	2.48	0.1327
**time*temp**	2	1891.987222	945.993611	1.99	0.1661
**ratio*temp**	2	1895.333889	947.666944	1.99	0.1657
**time*ratio*temp**	4	7331.276111	1832.819028	3.85	0.0198

DPPH activity dataset

Source	DF	Sum of Squares	Mean Square	F Value	Pr > F
**Model**	17	84790.8056	4987.6944	1.07	0.4397
**Error**	18	83595.5000	4644.1944		
**Corrected Total**	35	168386.3056			

Proanthocyanidin content dataset

Source	DF	Sum of Squares	Mean Square	F Value	Pr > F
**Model**	17	4088.830000	240.519412	1.36	0.2639
**Error**	18	3193.640000	177.424444		
**Corrected Total**	35	7282.470000			

Sugar content dataset

Source	DF	Sum of Squares	Mean Square	F Value	Pr > F
**Model**	17	54528.58250	3207.56368	3.12	0.0106
**Error**	18	18488.88500	1027.16028		
**Corrected Total**	35	73017.46750			

R-Square	Coeff Var	Root MSE	sucr Mean
0.746788	5.437391	32.04934	589.4250

Source	DF	Type I SS	Mean Square	F Value	Pr > F
**time**	2	2984.81167	1492.40583	1.45	0.2600
**ratio**	2	12953.50167	6476.75083	6.31	0.0084
**time*ratio**	4	15407.39167	3851.84792	3.75	0.0218
**temp**	1	230.53361	230.53361	0.22	0.6414
**time*temp**	2	5164.22056	2582.11028	2.51	0.1089
**ratio*temp**	2	1489.49389	744.74694	0.73	0.4979
**time*ratio*temp**	4	16298.62944	4074.65736	3.97	0.0177

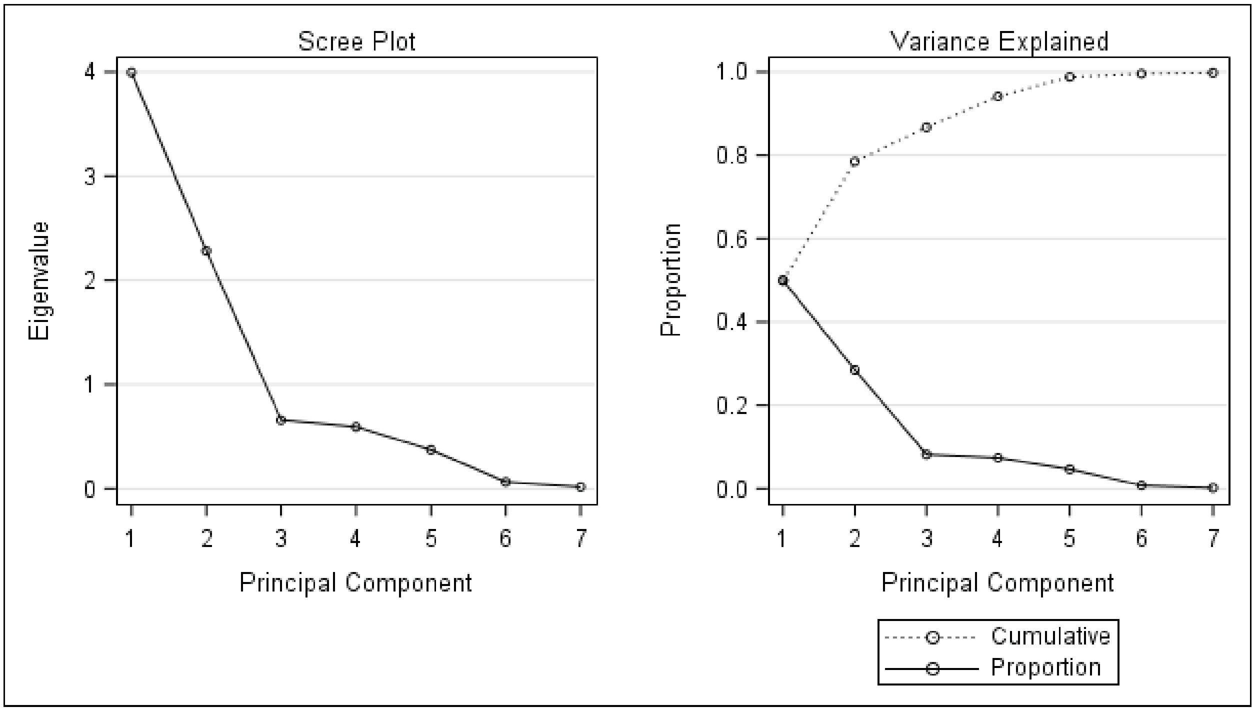

Figure S1: Variance explained for the principal component analysis

Table S2. Pearson correlation coefficients and p values from statistical significance testing (in italics).

Compound	PC1	PC2	PC3
1	0.14514<br>*0.3983*	0.96254<br>*0.6483*	0.07868<br>0.6483
2	0.28258<br>*0.0949*	0.93507<br>*<.0001*	0.14035<br>0.4142
3	0.72520<br>*<.0001*	-0.35307<br>*0.0347*	0.09313<br>0.5890
4	0.86412<br>*<.0001*	0.02864<br>*0.8683*	-0.42546<br>0.0097
5	0.89105<br>*<.0001*	-0.31833<br>*0.0585*	-0.03004<br>0.8619
6	0.62628<br>*<.0001*	-0.35908<br>*0.0315*	0.59194<br>0.0001
7	0.87583<br>*<.0001*	0.05693<br>*0.7416*	-0.25692<br>0.1304
8	0.81732<br>*<.0001*	0.15916<br>*0.3538*	0.15916<br>0.3538

Supplementary Material – NMR

NMR shifts (^{1}H and ^{13}C) of compounds 1, 2, 3, 4, 5, 7 and 8.

***trans-p*-coumaric acid *β*-D-glucopyranoside (1) :** ^{1}H NMR (500 MHz, CD_3OD) δ 7.64 (d, *J* = 15.9 Hz, H-7), 7.56 (d, *J* = 8.8 Hz, H-2/6), 7.13 (d, *J* = 8.8 Hz, H-3), 7.10 (d, *J* = 8.7, H-5), 6.38 (d, *J* = 15.9, H-8), 4.98 (dd, *J* =7.4, 5.3 Hz, H-1′), 3.91- 3.37 (Glucose: 3.91 (dd, *J* = 12.1, 2.2 Hz, H-6a′), 3.71 (dd, *J* = 12.0, 5.8 Hz, H-6b′), 3.51 – 3.45 (m, H-2′/3′/5′), 3.43 – 3.37 (m, H-4′)). ^{13}C NMR (126 MHz, CD_3OD) δ 169.3 (COOD), 159.4 (C-4), 144.5 (C-7), 129.3 (C-2, 6), 128.6 (C-1), 116.5 (C-3, 5), 116.1 (C-8), 100.4 (C-1′), 76.8 (C-5′), 76.5 (C-3′), 73.4 (C-2′), 69.9 (C4′), 61.0 (C6′).

***trans*-ferulic acid *β*-D-glucopyranoside (2):** ^{1}H NMR (500 MHz, CD_3OD) δ 7.62 (d, *J* = 15.9 Hz, H-7), 7.26 (d, *J* = 1.8 Hz, H-2), 7.19 (d, *J* = 8.4 Hz, H-5), 7.16 (dd, *J* = 8.4, 1.8 Hz, H-6), 6.41 (d, *J* = 15.9 Hz, H-8), 4.98 (d, *J* = 7.4 Hz, H-1′), 3.91 (s, CH_3), 3.71- 3.37 (Glucose: 3.70 (dd, *J* = 12.1, 5.3 Hz, H-6a′), 3.53 (dd, *J* = 9.2, 7.5 Hz, H-6b′), 3.50-3.38 (m, H-2′, 3′, 5′)). ^{13}C NMR (126 MHz, CD_3OD) δ 169.2 (COOD), 149.6 (C-3), 148.6 (C-4), 144.7 (C-7), 129.2 (C-1), 122.0 (C-6), 116.5 (C-8), 115.9 (C-5), 111.0 (C-2), 100.8 (C-1′), 76.9 (C-3′), 76.4 (C-5′), 73.4 (C-2′), 69.8 (C-4′), 61.0 (C-6′), 55.3 (CH_3).

***trans*-astringin (3):** ^{1}H NMR (500 MHz, DMSO-d_6) δ 6.97 (d, *J* = 2.1 Hz, H-10), 6.95 (d, *J* = 16.2 Hz, H-8), 6.84 (dd, *J* = 8.2, 2.1 Hz, H-14), 6.77 (d, *J* = 16.2 Hz, H-7), 6.72 (br d, *J* = 4.8 Hz, H-13), 6.71 (br s, H-2), 6.56 (t, *J* = 1.6 Hz, H-6), 6.33 (t, *J* = 2.2 Hz, H-4), 4.81 (d, *J* = 7.7 Hz, H-1′), 3.72-3.16 (Glucose: 3.72 (d, *J* =9.6 Hz, H-6a′), 3.55-3.16 (m, 5H)). ^{13}C NMR (126 MHz, DMSO-d_6) δ 159.3 (C-3), 158.8 (C-5), 146.1 (C-12), 145.8 (C-11), 139.8 (C-9), 129.4 (C-1), 128.9 (C-8), 125.5 (C-7), 119.2 (C-14), 116.1 (C-13), 113.8 (C-10), 107.5 (C-6), 105.2 (C-2), 103.2 (C-4), 101.1 (C-1′), 77.5 (C-5′), 77.1 (C-3′), 73.7 (C-2′), 70.2 (C-4′), 61.2 (C-6′).

***trans*-piceid (4):** ^{1}H NMR (500 MHz, DMSO-d_6) δ 7.39 (d, *J* = 8.1 Hz, H-10, 14), 7.02 (d, *J* = 16.4 Hz, H-8), 6.88 (d, *J* = 16.3 Hz, H-7), 6.75 (d, *J* = 8.1 Hz, H-13, 11), 6.72 (d, *J* = 5.5 Hz, H-2), 6.56 (br s, H-6), 6.33 (br s, H-4), 4.80 (d, *J* = 7.7, H-1′), 3.72– 3.13 (Glucose: 3.72 (d, *J* = 11.7 Hz, H-6a′), 3.48 (dd, *J* = 11.89, 5.8 Hz, H-6b′), 3.41-3.13, (m, 4H). ^{13}C NMR (126 MHz, DMSO-d_6) δ 159.3 (C-3), 158.8 (C-5), 157.8 (C-12), 147.2 (C-11), 139.8 (C-9), 129.0 (C-1), 128.4 (C-8), 125.6 (C-7), 120.7 (C-14), 116.0 (C-13), 110.3 (C-10), 107.6 (C-6), 105.1 (C-2), 103.2 (C-4), 101.1 (C-1′), 77.6 (C-3′), 77.2 (C-5′), 73.7 (C-2′), 70.2 (C-4′), 61.1 (C-6′).

***trans*-isorhapontin (5)** : ^{1}H NMR (500 MHz, DMSO-d_6) δ 7.17 (d, *J* = 2.0 Hz, H-10), 7.02 (d, *J* = 16.3 Hz, H-8), 6.96 (dd, *J* = 8.2, 2.0 Hz, H-14), 6.91 (d, *J* = 16.3 Hz, H-7), 6.75 (d, *J* = 8.1 Hz, H-13), 6.74 (t, *J* = 1.7 Hz, H-2), 6.56 (t, *J* = 1.7 Hz, H-6), 6.33 (t, *J* = 2.2 Hz, H-4), 4.80 (d,

J = 7.6 Hz, H-1'), 3.82 (s, CH3O), 5,30-3,16 (Glucose : 5.30 – 5.25 (m, OH-3'), 5.09 (s, OH-2'), 5.05 – 4.99 (m, OH-4'), 4.63 (t, *J* = 5.6 Hz, OH-6'), 3.72 (m, H-6a′), 3.48 (dd, *J* = 11.6, 5.6 Hz, H-6b'), 3.26 (d, *J* = 8.8 Hz, H-3'), 3.16 (d, *J* = 2.7 Hz, H-4'), 4.63 (t, *J* = 5.6 Hz, OH-6')). ^{13}C NMR (126 MHz, DMSO-d_6) δ 159.3 (C-3), 158.8 (C-5), 148.2 (C-11), 147.1 (C-12), 139.8 (C-9), 129.3 (C-1), 129.0 (C-8), 126.0 (C-7), 120.7 (C-14), 116.0 (C-13), 110.3 (C-10), 107.7 (C-6), 105.1 (C-2), 103.2 (C-4), 101.1 (C-1′), 77.6 (C-5′), 77.1 (C-3′), 73.7 (C-2′), 70.2 (C-4′), 61.2 (C-6′), 56.1 (CH_3O).

***trans*-resveratrol (7):** ^{1}H NMR (500 MHz, DMSO-d_6) δ 9.56 (s, OH-12), 9.20 (s, OH-3, OH-5), 7.39 (d, *J* = 8.5 Hz, H-10, H-14), 6.92 (d, *J* = 16.3 Hz, H-8), 6.81 (d, *J* = 16.3 Hz, H-7), 6.75 (d, *J* = 8.5 Hz, H-11, H-13), 6.38 (d, *J* = 2.2 Hz, H-2, H-6), 6.11 (t, *J* = 2.1 Hz, H-4). ^{13}C NMR (126 MHz, DMSO-d_6) δ 158.9 (C-3,5), 157.6 (C-12), 139.7 (C-9), 128.5 (C-1), 128.3 (C-10,14,8 overlapping), 126.1 (C-7), 115.9 (C-11, 13), 104.7 (C-2,6), 102.2 (C-4).

***trans*-isorhapontigenin (8):** ^{1}H NMR (500 MHz, DMSO-d_6) δ 7.17 (d, *J* = 2.0 Hz, H-10), 6.95 (dd, *J* = 8.2, 2.0 Hz, H-14), 6.92 (d, *J* = 16.0 Hz, H-8), 6.86 (d, *J* = 16.3 Hz, H-7), 6.74 (d, *J* = 8.0 Hz, H-13), 6.38 (d, *J* = 2.2 Hz, H-2, 6), 6.11 (t, *J* = 2.2 Hz, H-4), 3.82 (s, CH_3O). ^{13}C NMR (126 MHz, DMSO-d_6) δ 158.9 (C-3), 158.7 (C-5), 148.2 (C-11), 147.0 (C-12), 139.7 (C-9), 129.8 (C-1), 129.0 (C-8), 126.3 (C-7), 120.6 (C-14), 115.9 (C-13), 110.3 (C-10), 106.8 (C-6), 104.7 (C-2), 102.2 (C-4), 56.1 (CH_3O).

Figure S2. 1H NMR of compound 6.

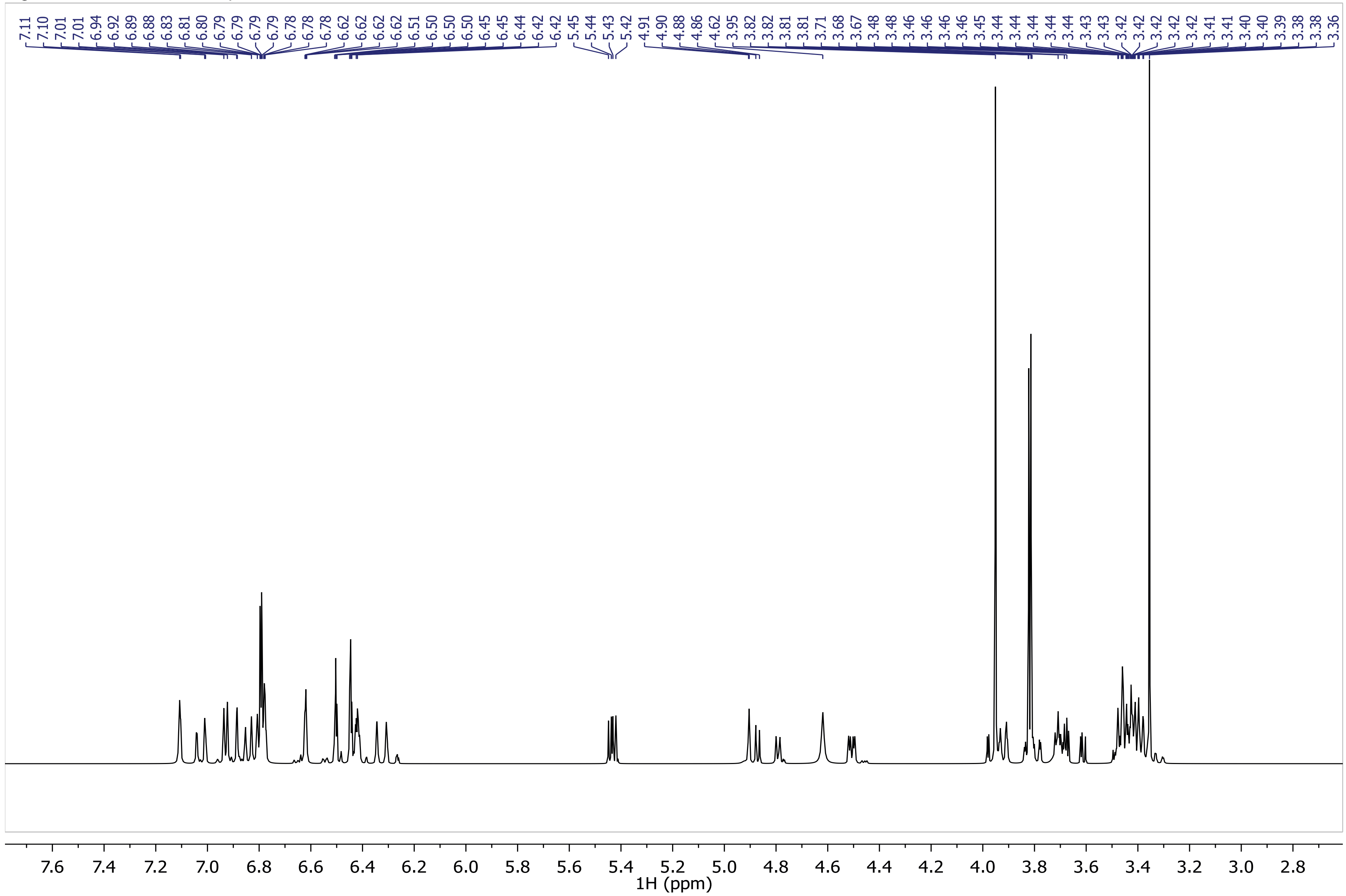

Figure S3. 13C NMR of compound 6.

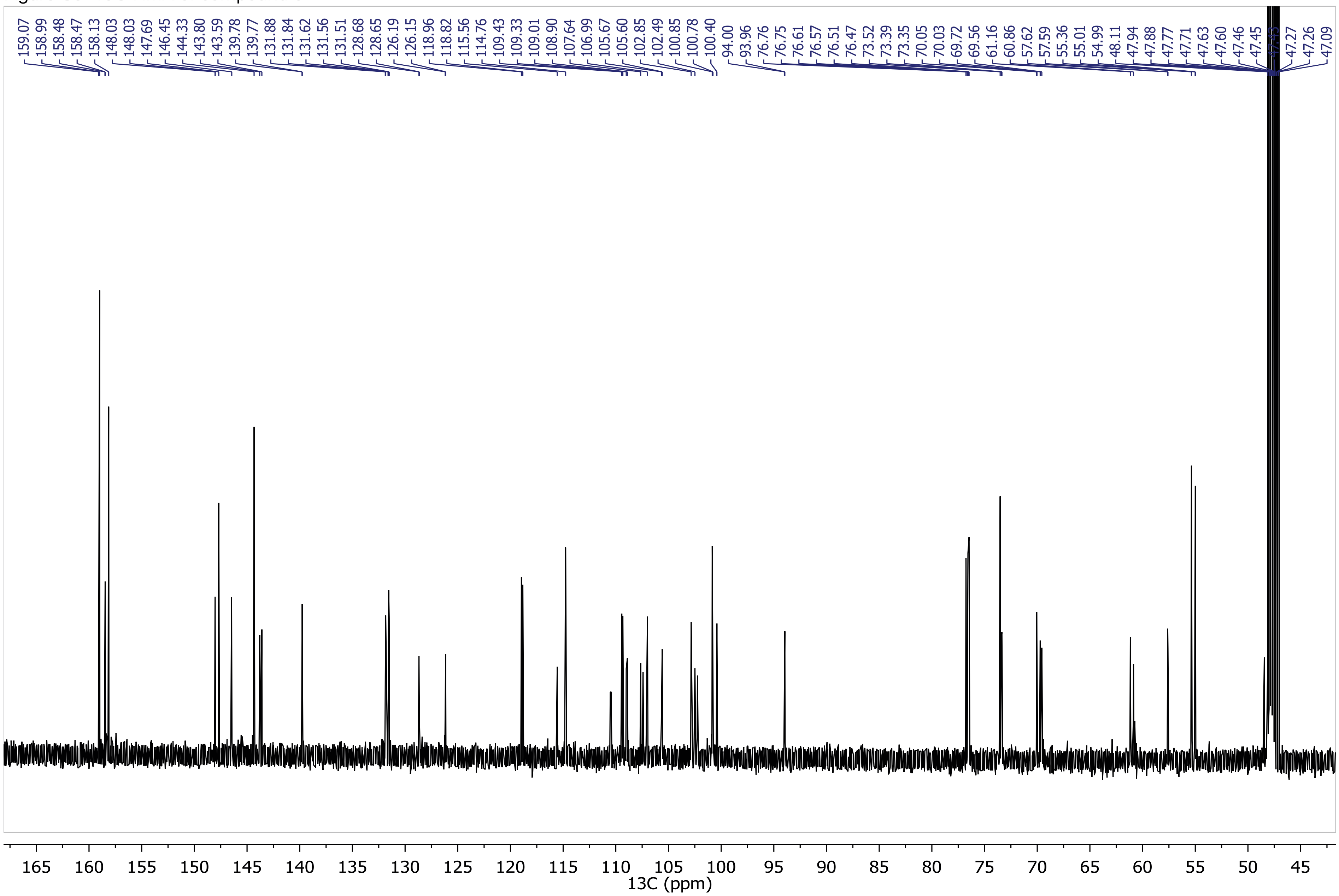

Figure S4. HSQC NMR of compound 6.

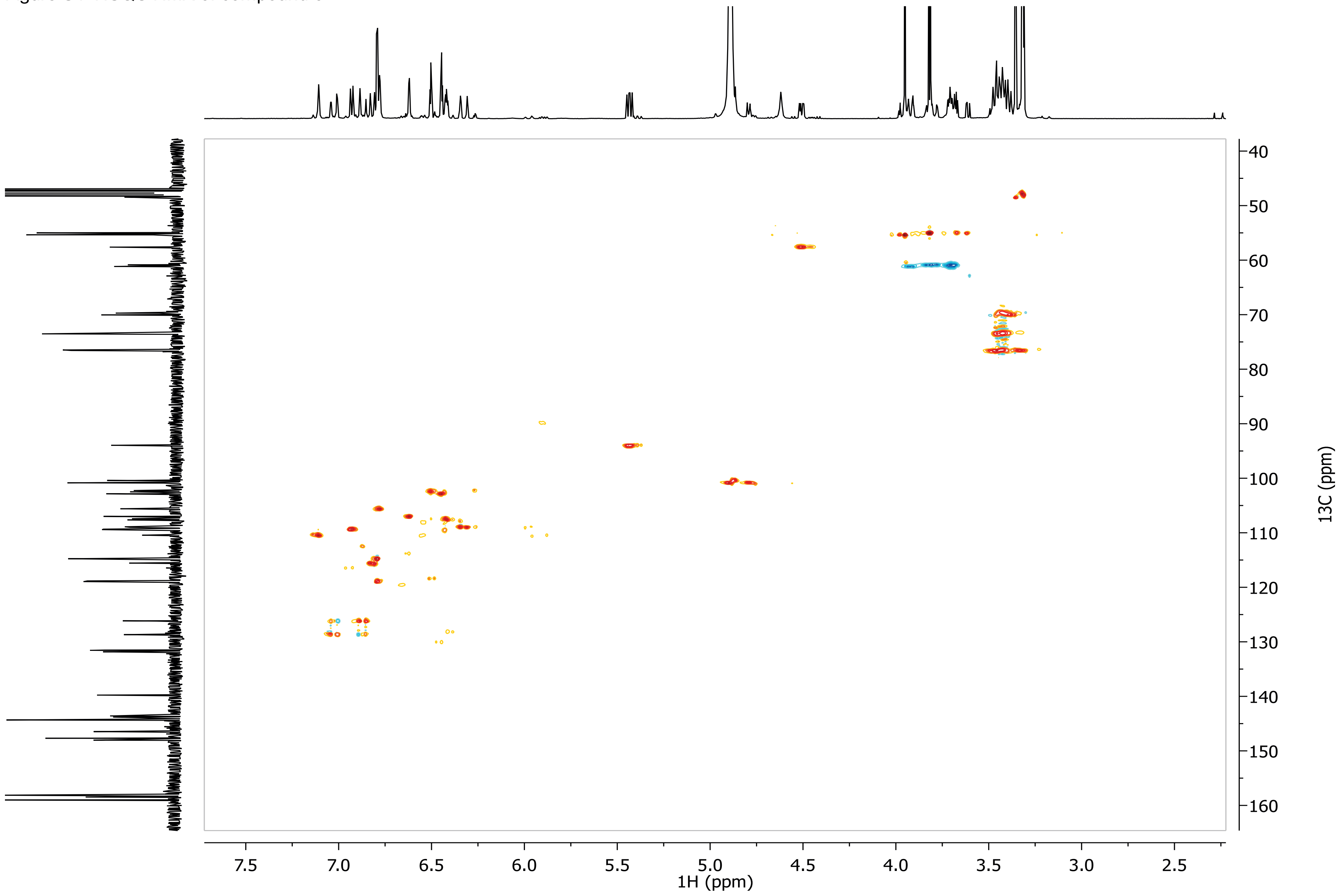

Figure S5. HMBC NMR of compound 6.

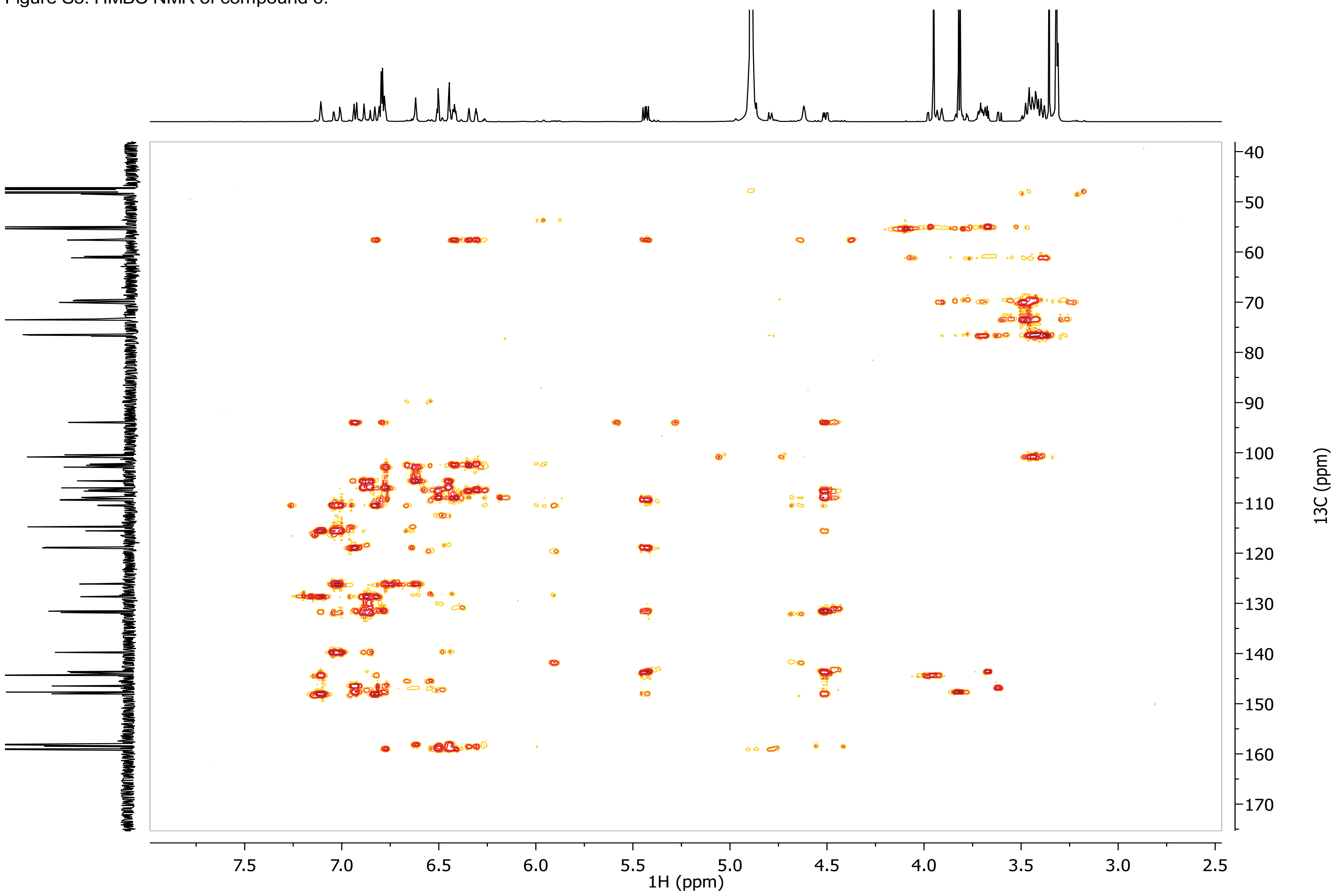

Supplement: Supplementary file 1 [file molecules-22-02118-s001.pdf]
